# Imbedding Pd Nanoparticles into Porous In_2_O_3_ Structure for Enhanced Low-Concentration Methane Sensing

**DOI:** 10.3390/s23031163

**Published:** 2023-01-19

**Authors:** Xiaoyang Zuo, Zhengyi Yang, Jing Kong, Zejun Han, Jianxin Zhang, Xiangwei Meng, Shuyan Hao, Lili Wu, Simeng Wu, Jiurong Liu, Zhou Wang, Fenglong Wang

**Affiliations:** 1 Key Laboratory for Liquid-Solid Structural Evolution and Processing of Materials Ministry of Education, Shandong University, Jinan 250061, China; 2 China Aerospace Components Engineering Center, China Academy of Space Technology, Beijing 100094, China

**Keywords:** indium oxide, MIL-68 (In), Pd nanoparticles, methane gas sensing, oxygen vacancy, catalytic effect

## Abstract

Methane (CH_4_), as the main component of natural gas and coal mine gas, is widely used in daily life and industrial processes and its leakage always causes undesirable misadventures. Thus, the rapid detection of low concentration methane is quite necessary. However, due to its robust chemical stability resulting from the strong tetrahedral-symmetry structure, the methane molecules are usually chemically inert to the sensing layers in detectors, making the rapid and efficient alert a big challenge. In this work, palladium nanoparticles (Pd NPs) embedded indium oxide porous hollow tubes (In_2_O_3_ PHTs) were successfully synthesized using Pd@MIL-68 (In) MOFs as precursors. All In_2_O_3_-based samples derived from Pd@MIL-68 (In) MOFs inherited the morphology of the precursors and exhibited the feature of hexagonal hollow tubes with porous architecture. The gas-sensing performances to 5000 ppm CH_4_ were evaluated and it was found that Pd@In_2_O_3_-2 gave the best response (R_a_/R_g_ = 23.2) at 370 °C, which was 15.5 times higher than that of pristine-In_2_O_3_ sensors. In addition, the sensing materials also showed superior selectivity against interfering gases and a rather short response/recovery time of 7 s/5 s. The enhancement in sensing performances of Pd@In_2_O_3_-2 could be attributed to the large surface area, rich porosity, abundant oxygen vacancies and the catalytic function of Pd NPs.

## 1. Introduction

As the main component of natural gas, liquefied gas, and coal mine gas, methane has gained large-scale application of in daily life and industrial processes [1,2]. It is noted that CH_4_ is inflammable and explosive at ambient temperature and pressure [3]. Once the concentration of CH_4_ reaches the explosive limit (4.9–15.4%) in a relatively airtight space, a severe explosion is likely to occur, resulting in serious casualties and property losses [4]. Furthermore, methane is also considered as a powerful greenhouse gas, of which the greenhouse effect reported by the Environmental Protection Agency (EPA) is about 20~25 times higher than that of CO_2_ [5,6]. What is worse, CH_4_ is a considerable threat to environment because it can remain for over 12 years in the atmosphere until complete decomposition [7]. Therefore, it is of great significance to develop cost-effective technique that can track methane in time so as to ensure the safety of the environment.

Methane is highly thermodynamic stable owing to its firm tetrahedral-symmetry structure with the bond energy of 431 kJ/mol [8]. Thus, it is distinctly an enormous challenge to detect methane efficiently. Gas sensors based on metal oxide semiconductors (MOSs), including SnO_2_ [9,10], ZnO [11,12], WO_3_ [2], VO_x_ [13], NiO [14,15], and In_2_O_3_ [16], have been widely studied to detect methane because of their high response, wide linear range, low detection limit, excellent anti-interference capacity, facile operation, and low cost [17]. As an n-type semiconductor with a wide band gap of 3.5~3.7 eV, In_2_O_3_, with various nanostructures such as nanosheets, nanoparticles, and nanospheres, has attracted considerable attention due to its superior electrical properties, showing potential to fabricate methane sensors for application [18,19,20,21]. However, pristine In_2_O_3_ sensors are still suffering from some drawbacks, such as low sensitivity, long-time response and recovery, as well as poor selectivity to methane. Therefore, it is crucial to enhance their methane-sensing performance by changing structure of the materials.

MOSs derived from metal-organic frameworks (MOFs) have been extensively applied in catalysis [22,23], battery [24] and gas sensors [25]. Moreover, MOFs offer great opportunities for accommodating myriad guest materials, including noble metal nanoparticles, and still remain the internal MOF structure for further application [26]. Their large specific surface area, regular porous morphologies, and tunable microstructures are convenient for gas diffusion [27,28]. Li et al. fabricated a productive H_2_S gas sensor based on the bamboo-like CuO/In_2_O_3_ derived from Cu^2+^-impregnated CPP-3 (In) with a low detection limit (200 ppb) at a low operating temperature (70 °C) [29]. Ma et al. successfully synthesized a chlorine gas sensor using MIL-68 (In) as template and the improved sensitivity and selectivity were obtained at 160 °C, which can be attributed to abundant oxygen vacancies and active sites [30]. Furthermore, modification with noble metals, especially palladium (Pd), has been proved to be an extremely effective approach to enhance the sensing properties of MOSs towards methane. Wang et al. prepared Pd-modified ZnO nanosheets, exhibiting not only appreciable response but also a declined optimum working temperature when the loading amount of Pd was 1 at% [31]. In addition, a reliable CH_4_ sensor based on Pd-loaded SnO_2_ hollow spheres was manufactured by Yang et al. and demonstrated a response value of 4.88 to 250 ppm CH_4_ at a decreased temperature of 300 °C [32]. Based on these pioneer works, it is possibly a marvelous idea to combine the large surface area of In_2_O_3_ MOSs using metal-organic frameworks as template, with the catalytic effect of Pd NPs in order to improve the methane sensing performance. Nonetheless, there is still no relevant literature reported constructing methane gas sensors based on Pd@In_2_O_3_ derived from Pd@MIL-68 (In) precursors with core-shell structure.

In this work, Pd@In_2_O_3_ porous hollow tubes (PHTs) were successfully synthesized through a facile solvothermal method combined with subsequent calcination and reduction. Along with the construction of MIL-68 (In) prisms, different amounts of Pd NPs were injected to form the core-shell structure, ensuring intimate interaction between Pd NPs and MOFs. The sensing performance towards 5000 ppm CH_4_ of all prepared sensors were investigated and compared. We found that Pd@In_2_O_3_-2 sensor could be a promising candidate for methane detection. The mechanism for the enhanced methane-sensing performance of the materials were thoroughly discussed.

## 2. Experimental Section

### 2.1. Materials

1,4-benzenedicarboxylic acid (H_2_BDC), N,N-dimethylformamide (DMF), polyvinylpyrrolidone K30 (PVP K30), potassium iodide (KI), and formamide (FA) were acquired from Sinopharm Chemical Reagent Co., Ltd., Shanghai, China. Palladium chloride (PdCl_2_) and indium nitrate hydrate (In(NO_3_)_3_·xH_2_O) were purchased from Shanghai Macklin Biochemical Co., Ltd., Shanghai, China. All the chemicals and reagents were of chemical purity and used as received.

### 2.2. Preparation of Pd Nanoparticles Suspension, Porous Hollow In_2_O_3_ Tubes and Pd@In_2_O_3_ Porous Hollow Tubes

The synthesis of Pd nanoparticles was carried out by modifying the previously reported method [33]. First, 50 mg PVP K30 and 170 mg KI were dissolved into 5 mL FA under vigorously stirring and the mixture was heated in oil bath until its temperature reached 120 °C. Afterwards, 36 mg PdCl_2_ was rapidly added into the solution and the temperature maintained at 120 °C for 1 h. Ultimately, the Pd nanoparticles suspension was obtained and used without any other processing.

The MIL-68 (In) precursors were synthesized by a modified solvothermal method [34]. In a typical process, 0.27 g In(NO_3_)_3_·xH_2_O and 0.33 g H_2_BDC were dissolved in 120 mL DMF. After being stirred for 15 min, the transparent solution was subject to be heated at 120 °C for 45 min in an oil bath. Subsequently, the obtained white precipitate was filtrated and washed with DMF and ethanol three times, respectively, and dried at 60 °C overnight to obtain MIL-68 (In) precursors. Ultimately, the porous In_2_O_3_ nanorods were acquired through calcination of the precursors at 500 °C for 50 min with a ramping rate of 5 °C/min in a muffle furnace.

**Figure 1:**
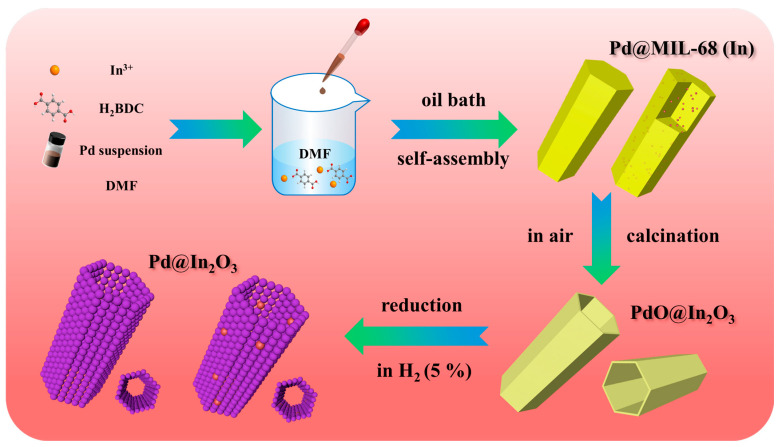
Schematic illustration of the preparation process of Pd@In_2_O_3_ PHTs.

The fabrication process of Pd@In_2_O_3_ is schematically illustrated in Figure 1. Typically, a certain volume of Pd NPs suspension (0.63 mL, 1.26 mL, and 1.88 mL) was rapidly injected into the solution of In(NO_3_)_3_·xH_2_O and H_2_BDC at 120 °C and maintained for 45 min to obtain the Pd@MIL-68 (In) rods. Afterwards, the Pd@MIL-68 (In) precursors were calcined in a muffle furnace at 500 °C for 50 min in air to gain the PdO@In_2_O_3_ hollow tubes. Eventually, the Pd@In_2_O_3_ PHTs were acquired by reducing PdO@In_2_O_3_ using H_2_ (5 vol.% in N_2_) at 500 °C for 2 h with a temperature ramp of 2 °C/min in the tube furnace. The as-prepared Pd@In_2_O_3_ PHTs were denoted as Pd@In_2_O_3_-1, Pd@In_2_O_3_-2, Pd@In_2_O_3_-3, respectively corresponding to the contents of Pd NPs.

### 2.3. Characterizations

The crystal structure was characterized by the X-ray diffraction (XRD, DMAX-2500PC) instrument with Cu-Kα radiation (λ = 1.5418 Å) in the range of 10°–90°. In order to observe the morphology and microstructure of the fabricated samples, a SU-70 field emission scanning electron microscope (FE-SEM) with an accelerating voltage of 15 kV, a JEM-1011 transmission electron microscopy (TEM) with an accelerating voltage of 100 kV and a JEM-2100 high-resolution transmission electron microscopy (HR-TEM) with an accelerating voltage of 200 kV were employed. X-ray photoelectron spectroscopy (XPS, Thermo Scientific ESCALAB Xi+, Waltham, MA, USA) using a monochromatic Al-Kα radiation resource (hʋ = 1486.6 eV) was implemented. The Electron Paramagnetic Resonance (EPR) spectra were recorded using a Bruker A300 at 9.85 GHz at 77 K. Room temperature UV–vis diffuse reflectance spectra of In_2_O_3_ and Pd@In_2_O_3_ were recorded on a Shimadzu UV-3600Plus ultraviolet spectrophotometer.

### 2.4. Sensing Performances Test

**Figure 2:**
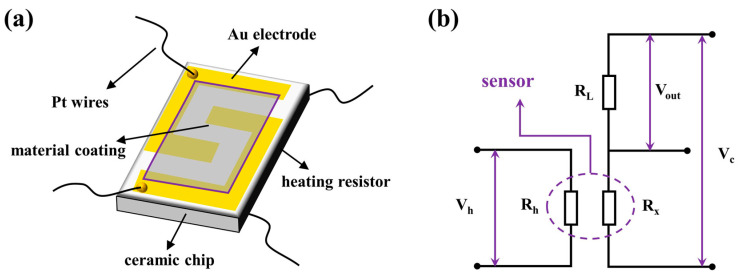
(**a**) Schematic diagram of gas sensor element and (**b**) the sensor testing principle.

The gas-sensitivity sensors based on prepared sensing material were fabricated as follows. Generally, the synthesized materials were mixed with a mixture of terpineol and ethyl cellulose (M70) with a fixed weight ratio of 1:99, and then the mixture was ground until a uniform slurry was obtained. Afterwards, the paste was uniformly coated on the Al_2_O_3_ ceramic substrate with a size of 2 mm in length and 1 mm in width, equipped with a pair of Au electrodes and four Pt wires, as graphically illustrated in Figure 2a. After that, the substrates smeared with sensing materials were calcined at 400 °C for 2 h in air. Ultimately, the substrates were welded on pedestals to gain gas sensors. The gas sensors were aged with a voltage of 5 V added across for about 7 days to stabilize the electrical properties before tested. The gas-sensing properties were evaluated on a WS-30A gas sensitivity instrument (Zhengzhou Winsen Electronics Co., Ltd., Zhengzhou, China) at 25 °C without air humidity exceeding 30%. The concentrations of methane were regulated by adjusting the volume of methane and dry air injected into the glass chamber. The testing system is illustrated in Figure 2b, in which the R_h_, R_x,_ and R_L_ are heating resistance, material resistance, and load resistance, respectively. Generally, adjustment of operating temperatures is realized by tuning the voltage (V_h_) applied across the R_h_. Meanwhile, a circle voltage (V_c_ = 5 V) is supplied across the sensing material and load resistance. The output electric signal is the voltage (V_out_) across the load resistor. Furthermore, the resistance of the sensing material could be expressed by the formula R_x_ = (V_c_ − V_out_) R_L_/V_c_. The sensitivity (S) of the reducing gas can be defined as S = R_a_/R_g_, where R_a_ and R_g_ are resistances of sensing material in air and methane, respectively. In addition to the sensitivity, the optimal operating temperature, response and recovery time, long-term stability, as well as selectivity are also evaluated as they are significant indicators to evaluate the sensing performance of a methane gas sensor [35]. The response or recovery time is well-defined as the time for a sensor required to achieve 90% of the whole resistance change when methane is input or output.

## 3. Results and Discussion

### 3.1. Characterizations

**Figure 3:**
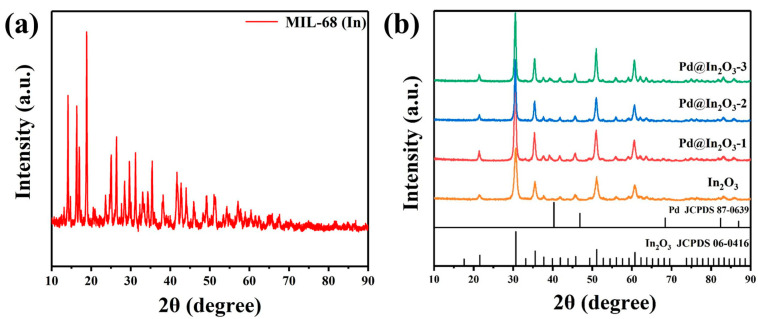
XRD patterns of (**a**) MIL-68 (In) and (**b**) pristine In_2_O_3_, Pd@In_2_O_3_-1, Pd@In_2_O_3_-2 and Pd@In_2_O_3_-3.

The phase structure of the fabricated sensing materials was investigated via XRD. Figure 3a shows the XRD pattern of prepared precursors, which agrees well with the previously reported data [34], confirming the successful synthesis of MIL-68 (In) precursors. Meanwhile, XRD patterns of pristine In_2_O_3_ and Pd@In_2_O_3_ with various Pd loadings are shown in Figure 3b. For the pristine In_2_O_3_, it can be observed that all diffraction peaks can be well indexed to cubic structure of In_2_O_3_ (JCPDS card No. 06-0416). As for Pd@In_2_O_3_ samples, there is no obvious Pd signal observed, and this can be chiefly attributed to the high dispersion of Pd with low loadings.

**Figure 4:**
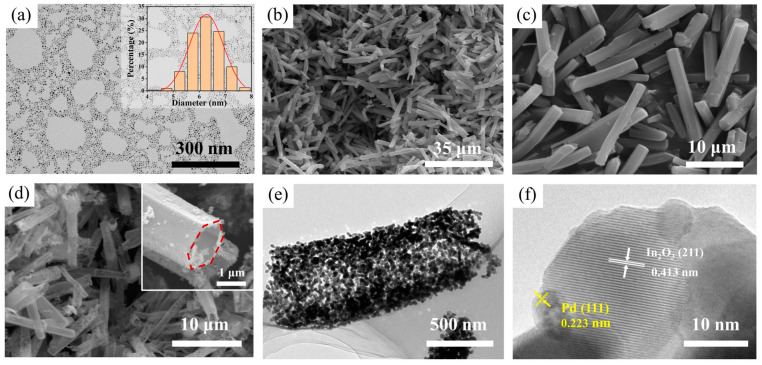
(**a**) TEM images of Pd nanoparticles (the inset is the size distribution histograms); SEM images of (**b**,**c**) MIL-68 (In) precursors, and (**d**) pristine In_2_O_3_ (the inset is the enlargement of the cross section of In_2_O_3_); (**e**,**f**) HRTEM images of Pd@In_2_O_3_-2.

The morphologies of prepared samples are displayed in Figure 4. As illustrated in Figure 4a, the TEM image demonstrates that the size of prepared Pd NPs ranges from 4 to 8 nm. As can be observed in Figure 4b,c, MIL-68 (In) precursors are solid hexagonal prisms with smooth surface, and the length of them varies from approximately 5 to 18 μm and the diameter is about 2 μm. After the calcination at 500 °C, the solid hexagonal rods transformed into the hollow microstructure with rough surface and porous shell, almost maintaining the geometric characteristics of MIL-68 (In) rods, which is illustrated in Figure 4d and the inset image. Due to the Kirkendall effect, the transformation can be attributed to the inter-diffusion between In ions and O ions [36]. To further investigate the morphology and microstructure of Pd@In_2_O_3_-2, TEM and HRTEM images are taken as shown in Figure 4e,f. It is obvious that Pd@In_2_O_3_-2 PHTs are composed of abundant small nanoparticles, which is accordant with the observation from SEM images. Figure 4f reveals the lattice fringes, among which the lattice spacings of 0.223 nm and 0.413 nm correspond with the (111) plane of Pd and (211) plane of In_2_O_3_, respectively, implying the successful hybridization of Pd nanoparticles into In_2_O_3_.

**Figure 5:**
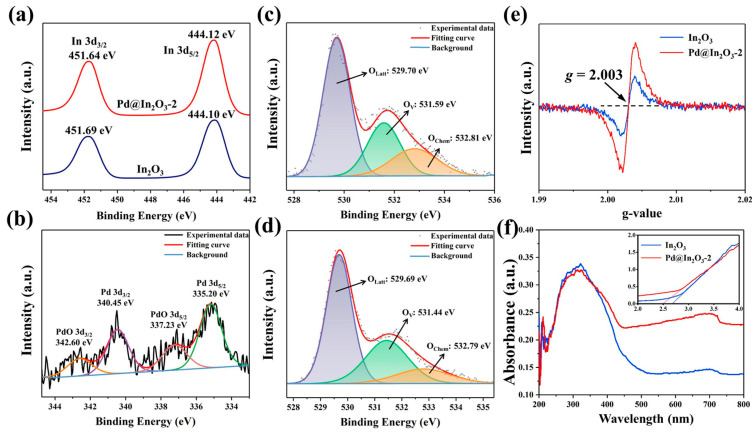
XPS spectra of the elements: (**a**) In 3d of pristine In_2_O_3_ and Pd@In_2_O_3_-2, (**b**) Pd 3d of Pd@In_2_O_3_-2, O 1s of (**c**) pristine In_2_O_3_ and (**d**) Pd@In_2_O_3_-2; characterization of oxygen vacancies: (**e**) electron paramagnetic resonance spectra, (**f**) UV-vis absorbance spectra, the inset is curves of (αhν)^2^ vs photo energy (hν).

The surface properties of pristine In_2_O_3_ and Pd@In_2_O_3_-2 were characterized by XPS. In the In 3d spectra shown in Figure 5a, the peaks at 444.1 eV and 451.69 eV in pristine In_2_O_3_ and the peaks at 444.12 eV and 451.64 eV in Pd@In_2_O_3_-2 correspond to In 3d_5/2_ and In 3d_3/2_ orbitals of In^3+^, respectively [37,38]. The XPS spectra of Pd 3d orbitals illustrated in Figure 5b signify that the two characteristic peaks situated at ca. 335.2 eV (Pd 3d_5/2_) and ca. 340.45 eV (Pd 3d_3/2_), with an energy interval of 5.25 eV are related to Pd^0^ [39]. Compared with the previous report [40], a shift of 0.3 eV towards the lower binding energy is observed, which is primarily triggered by the charge transfer between In_2_O_3_ and Pd NPs due to their different work functions. The peaks located at 337.23 eV and 342.6 eV are associated with Pd^2+^, indicating a small amount of PdO [41], mainly owing to the inevitable oxidation during storage.

To study the presence and content of oxygen species in pristine In_2_O_3_ and Pd@In_2_O_3_-2 PHTs, XPS spectra of O 1s orbital were analyzed. In Figure 5c,d, three deconvoluted peaks can be observed at ca. 529.8 eV, ca. 531.5, and ca. 532.7 eV, corresponding to the lattice oxygen (O_latt_), the surface oxygen vacancies (O_v_), and chemically absorbed oxygen species (O_chem_)(O^−^, O^2−^ and $O_{2}^{-}$), respectively [38,42]. The contents of the surface oxygen vacancies in pristine In_2_O_3_ and Pd@In_2_O_3_-2 are estimated to be 25.78% and 31.01%, respectively, presenting an increase in surface oxygen vacancies after the addition of Pd NPs. The increase of the defect was further confirmed by EPR. The *g* factor which is corresponding to ionized O_v_ is calculated to be 2.003, as shown in Figure 5e. The signal intensity of g value is correlated with the amount of oxygen defect, thus more oxygen vacancies can be observed in the Pd@In_2_O_3_-2 sample, which is consistent with the results of XPS. The augment of oxygen vacancies could make a positive effect on methane sensing performance. On one hand, the O_v_ species can provide additional active sites for reactants [43,44]. On the other hand, as electron donors, the increased oxygen vacancies enlarge the number of free electrons in the conduction band, thereby elevating the electronic mobility. In order to investigate the effect of the improvement of the defect on the energy band gap, UV-vis spectra were recorded in Figure 5f. The band gaps were calculated based on the Tauc model by the formula (αhν)^n^ = A (hν − E_g_), where α, h, ν, A, and E_g_ are the adsorption coefficient, Planck constant, light frequency, a material-dependent constant, and the band gap, respectively. For In_2_O_3_-based materials, n was chosen to be 2 [45]. As shown in the inset of Figure 5f, the band gaps are 2.69 eV and 2.50 eV for pristine In_2_O_3_ and Pd@In_2_O_3_-2, respectively. As shallow donor states under the conduction band, oxygen vacancies in metal oxide semiconductor could narrow the forbidden band gap. Hence, the improvement results in decrease of the forbidden gap width, so as to lower the energy barrier of methane adsorption and adjust the electronic structure of In_2_O_3_-based material, which is conducive to enhancing the sensitivity [46,47].

### 3.2. Gas-Sensing Performances towards Methane

Generally speaking, it is laborious to detect methane at low temperatures due to their high chemical thermal stability [15,48,49]. Thus, the working temperature should be elevated to increase the atomic activity of the surface between the material and methane molecules. For a gas sensor, there is an optimum operating temperature (O. T.) when the sensor exhibits the most outstanding sensitivity. A lower optimal operating temperature is expected to consume less energy, prolong the service life of sensor devices, and ensure the operation safety. The operating temperature has great impact on the reaction kinetics of the gas-sensing process, thus significantly influence the sensing performance [50].

**Figure 6:**
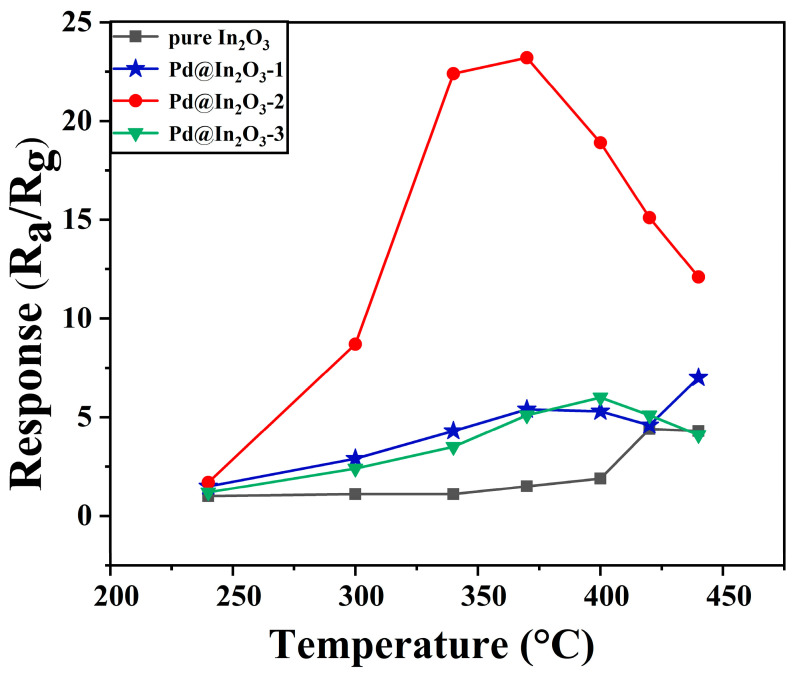
Gas response of pristine In_2_O_3_, Pd@In_2_O_3_-1, Pd@In_2_O_3_-2, and Pd@In_2_O_3_-3 to 5000 ppm CH_4_ at different temperatures.

In order to confirm the optimal operating temperature of the sensors, responses of all the fabricated sensors towards 5000 ppm methane at different temperatures were tested and recorded. As shown in Figure 6 and Table 1, a typical volcanic shape is observed in all samples. Originally, the sensitivity of the sensors towards methane is improved with the working temperature rising mainly because of the increased surface reaction kinetics. However, further increasing the temperature could not bring in the growth of responsibility. It is observed that the optimal operating temperature of Pd@In_2_O_3_-2 is 370 °C, which is 50 °C lower than that of the pristine In_2_O_3_ sensor (420 °C). Moreover, as shown in Figure 6 and Table 1, the Pd@In_2_O_3_-2 sensor exhibits the highest selectivity (S = 23.2) among all the samples at the optimum operating temperature of 370 °C, approximately 15.5 times higher than that of In_2_O_3_ sensor. When the response reaches the maximum, gas desorption assumes the predominance of the sensing reaction and restrains the sensing performance because of the higher temperature. The methane sensing performances of the Pd@In_2_O_3_-2 were further studied at the optimal operating temperature.

**Figure 7:**
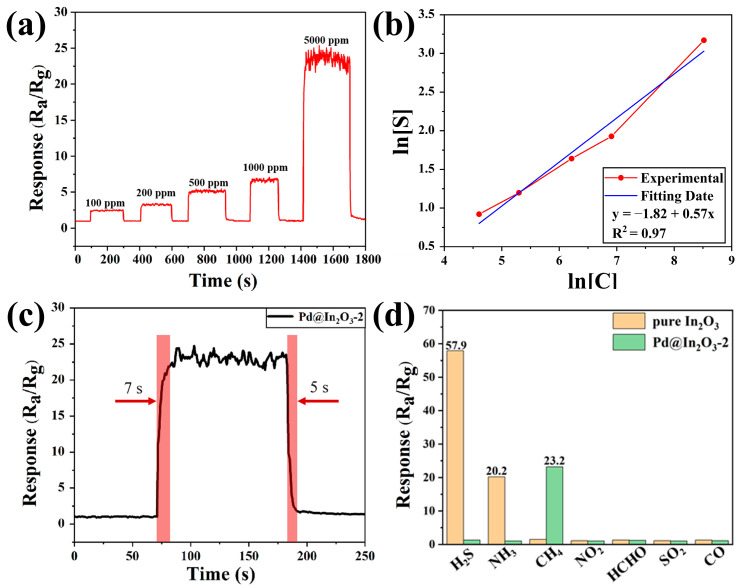
(**a**) Responses of Pd@In_2_O_3_-2 towards different concentrations of methane; (**b**) the linear fitting between ln[C] and ln[S]; (**c**) response and recovery time of Pd@In_2_O_3_-2 when exposed in 5000 ppm methane; (**d**) responses of pure In_2_O_3_ and Pd@In_2_O_3_-2 to 5000 ppm methane and other interfering gases.

High sensitivity is not the only criterion for evaluation of the gas sensors. The real-time kinetic curve of Pd@In_2_O_3_-2 sensors towards CH_4_ at 370 °C was further investigated and displayed in Figure 7a. The response gradually increases with the concentration of CH_4_ rising from 100 to 5000 ppm. Even when the concentration of methane is as low as 100 ppm, an obvious response value of 2.5 is observed. Figure 7b reveals that an evident linear correlation can be fitted as *y* = −1.82 + 0.57*x* between log S and log C, where S is the response and C is the concentration of methane. The satisfying linear fitting makes the quantitative detection of methane possible, signifying the potential application in device manufacture. According to the Martins-Naes theory for multivariate calibration, the detection limit (DL) can be extrapolated by the empirical formula as DL = 3 rms/slope, where rms is the root-mean-square deviation of the sensor noise calculated from the photocurrent signals in ambient air and the slope is obtained from the linear function [51]. Additionally, the real-time response curve of the Pd@In_2_O_3_-2 sensor to 5000 ppm CH_4_ is illustrated in Figure 7c, demonstrating a high-speed response and recovery. Its response and recovery time exposed to 5000 ppm methane is 7 s and 5 s, respectively. The fast response/recovery times are basically assigned to the efficient catalytic effect of Pd nanoparticles, which accelerates the reaction between adsorbed methane molecules and the oxygen species on the surface of sensors.

Selectivity is another crucial aspect to assess gas sensing properties. The response of pure In_2_O_3_ and Pd@In_2_O_3_-2 towards various interfering gases, such as nitrogen dioxide (NO_2_, 1 ppm), formaldehyde (HCHO, 1 ppm), sulfur dioxide (SO_2_, 5 ppm), carbon monoxide (CO, 24 ppm), sulfureted hydrogen (H_2_S, 40 ppm), and ammonia (NH_3_, 40 ppm), were investigated, which is depicted in Figure 7d. The selection of the concentrations of interfering gases is confirmed by the latest edition of coal mine security regulations for reference [15,52]. Compared with the extremely low response to CH_4_, pristine In_2_O_3_ performed higher responses to some interfering gases, such as H_2_S (57.9) and NH_3_ (20.2). However, it is observed that the responses of Pd@In_2_O_3_-2 to interferential gases are pretty weak (approximately 1) after adding moderate Pd NPs, which are much lower than that to methane (23.2). It is generally acknowledged that the value of response (R_a_/R_g_ > 2) is usually chosen as the standard for a valid response and thus the influence of interfering gases can be neglected in this work [29]. The modest introduction of Pd NPs promote not only the performance towards methane, but also the ability to resist interference of both H_2_S and NH_3_.

**Figure 8:**
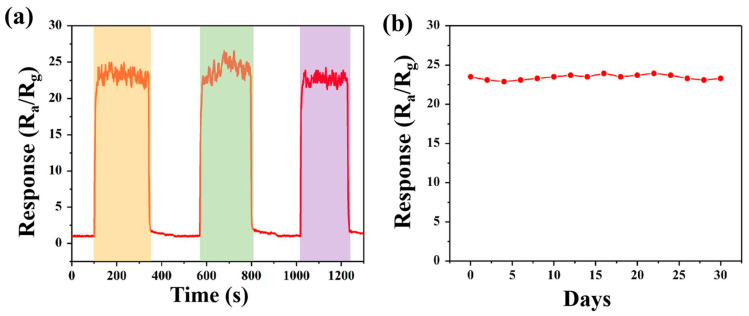
(**a**) The repeatability of Pd@In_2_O_3_-2 to 5000 ppm CH_4_; (**b**) long-term stability of Pd@In_2_O_3_-2 sensor towards 5000 ppm CH_4_ for 30 days.

Repeatability is another essential factor to evaluate the performances of gas sensors. As depicted in Figure 8a, the almost identical responses of Pd@In_2_O_3_-2 exposed to 5000 ppm methane based on three-times measurements demonstrate the satisfactory repeatability of Pd@In_2_O_3_-2. Furthermore, as shown in Figure 8b, the response maintains relatively stable during the one-month test, demonstrating an excellent long-term stability. In this work, the Pd@In_2_O_3_-2 based sensor exhibits prominent sensing properties and is highly competitive in methane detection. The improved sensing performance can be attributed to the unique porous morphology of hexagonal hollow tubes, as well as abundant oxygen vacancies originating from the thermal decomposition of MIL-68 (In) precursors and the existence of Pd NPs. Table 2 compares the methane sensing performances of Pd@In_2_O_3_-2 in this work with those in other literatures.

### 3.3. Gas-Sensing Mechanism

**Figure 9:**
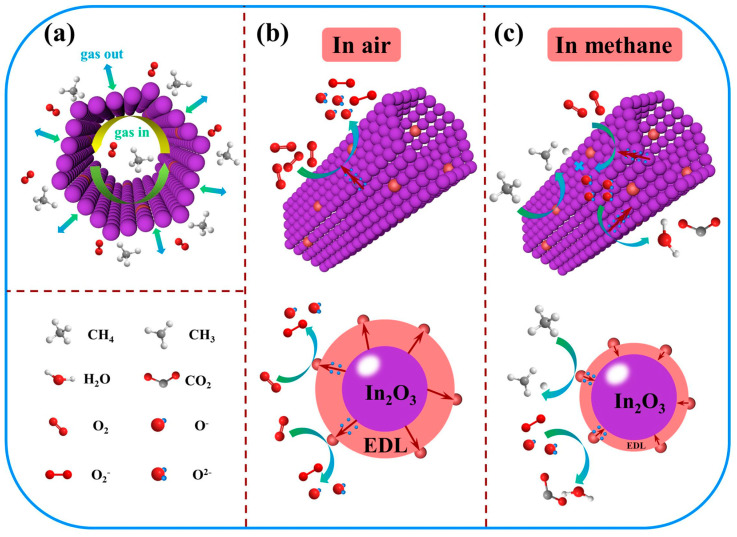
(**a**) The illustration of gas diffusion; Schematic diagram of methane sensing mechanism for Pd@In_2_O_3_-2 PHTs exposed to (**b**) air and (**c**) methane.

The porous, hollow, rod-like structure with large specific surface area inherited from MIL-68 (In) precursors provides abundant channels for methane molecules to diffuse in the sensing layer and affluent active sites for reaction, which is illustrated in Figure 9a. The surface adsorbed oxygen model is widely recognized as the gas-sensing mechanism, and the detailed interpretation is as follows. During gas adsorption-desorption process, electronic concentration in conduction band will make a huge difference on sensor resistance. First, when the sensing materials are exposed to the air, oxygen is physically adsorbed on the surface of the sensing materials without electron transfer during this process. With temperature gradually elevated, oxygen molecules capture electrons from the conduction band, forming chemisorbed oxygen species such as $O_{2}^{-}$, O^−^, and O^2−^ on the surface. Meanwhile, the thickness of electron depletion layer (EDL) increases, resulting in an increase in resistance. This process can be elaborated as follows [59,60]:

O_2gas_ → O_2ads_, (1)

(2) $$O_{2\mathrm{ads}}+e^{-}\to {O_{2}^{-}}_{\mathrm{ads}}$$

(3) $${O_{2}^{-}}_{\mathrm{ads}}+e^{-}\to 2\;{O^{-}}_{\mathrm{ads}}$$

O^−^_ads_ + e^−^ → O^2−^_ads_. (4)

Indium oxide (In_2_O_3_) is a typically n-type semiconductor metal oxide. Once In_2_O_3_ encounters with methane, the reducing gas will react with the active oxygen ions ($O_{2}^{-}$, O^−^, and O^2−^) and generates CO_2_ and H_2_O with electrons releasing back to the conduction band. Consequently, the thickness of EDL and resistance tend to descend. Therefore, measuring the resistance in air and methane atmosphere is an efficient method to monitor the CH_4_ in real time [61]. The process is shown as Equations (5) and (6) [62]:

CH_4gas_ + 4 O^−^_ads_ → CO_2gas_ + 2 H_2_O_gas_+ 4 e^−^, (5)

CH_4gas_ + 4 O^2−^_ads_ → CO_2gas_ + 2 H_2_O_gas_ + 8 e^−^. (6)

In this work, the improved sensing performance can be mainly attributed to the catalytic effect of Pd nanoparticles. The schematic diagram of methane sensing mechanism for Pd@In_2_O_3_-2 PHTs in air and methane was shown in Figure 9b.

On one hand, the Fermi levels of noble metals usually lie below the conduction band of semiconductor metal oxide, and the work function of Pd (W_m_ = 5.60 eV) is higher than that of In_2_O_3_ (W_s_ = 5.0 eV) [63,64]. Thus, the energy bands of In_2_O_3_ and Pd on contact surface will bend until the Fermi energy levels reach the equilibrium. The change of the Fermi level will promote the electrons to transfer from the surface of In_2_O_3_ to Pd NPs, motivating the sensing material to perform enhanced performance. On the other hand, the catalytic effect of Pd NPs effectively activates dissociation of oxygen molecules, which will flow over from the complex and diffuse on the surface of In_2_O_3_, capturing electrons from the metal oxide [65]. This process increases the concentration of adsorbed oxygen ions and makes the depletion layer wider, providing more active sites for the adsorption reaction of methane [66]. Moreover, when the sensing material is exposed to methane, Pd will facilitate the dissociation of C–H bond to form H and CH_3_, which spill over to the surface and react with chemically adsorbed oxygen ions on the surface of sensitive film, generating water molecules. During this process, the trapped electrons release back to the conduction band of In_2_O_3_, thus the thickness of EDL and the resistance of Pd@In_2_O_3_ interface decreases, reducing the barrier height of reaction [67]. The specific reactions on the surface of Pd@In_2_O_3_-2 are as follows:

CH_4_ → CH_3ads_ + H_ads_, (7)

CH_3_ + H + 4 O^2−^ → CO_2_ + 2 H_2_O + 8 e^−^. (8)

## 4. Conclusions

In this work, Pd@In_2_O_3_ porous hollow tubes derived from Pd@MIL-68 (In) precursors with core-shell structure were successfully synthesized through a hydrothermal method with subsequent calcination and reduction processes. Among all prepared samples, Pd@In_2_O_3_-2 exhibited the best performance in methane detection. With a response value of 23.2 at a relatively lower working temperature (370 °C), the sensitivity of Pd@In_2_O_3_-2 towards 5000 ppm methane was about 15.5 times higher than that of pristine In_2_O_3_, and the response and recovery times were only 7 s and 5 s, respectively. Furthermore, Pd@In_2_O_3_-2 PHTs also enable to selectively track methane with the presence of interfering gases, exhibiting an outstanding anti-interference capacity. Thus, this research indicates that the porous Pd@In_2_O_3_ are promising candidates for fabrication of methane sensors due to the large specific surface area, porous microstructure, increased oxygen vacancies and the catalytic effect of Pd nanoparticles.

## Figures and Tables

**Table 1:** Response of pristine In_2_O_3_ and Pd@In_2_O_3_-1, Pd@In_2_O_3_-2, and Pd@In_2_O_3_-3 samples at different temperatures.

Sample	240 °C	300 °C	340 °C	370 °C	400 °C	420 °C	440 °C
In_2_O_3_	1.0	1.1	1.1	1.5	1.9	4.4	4.3
Pd@In_2_O_3_-1	1.5	2.9	4.3	5.4	5.3	4.6	7.0
Pd@In_2_O_3_-2	1.7	8.7	22.4	23.2	18.9	15.1	12.1
Pd@In_2_O_3_-3	1.2	2.4	3.5	5.1	6.0	5.1	4.1

**Table 2:** Comparison of methane gas sensing properties of different materials from recent publications.

Sensing Materials	Sensitivity	O. T. (°C)	Concentration (ppm)	T_res_/T_rec_ (s)	Ref.
SnO_2_	1.25	400	1000	14/37	[53]
PdPt/SnO_2_	5.2	320	1000	5/4	[54]
Pt/SnO_2_	2.13	325	5000	-/-	[55]
SnO_2_-Pd	20	400	6600	-/-	[56]
Pt-SnO_2_	4.5	350	1000	30/150	[57]
In_2_O_3_ film	2.25	300	10,000	200/-	[16]
In_2_O_3_ spheres	6.50	350	5000	-/-	[58]
Pd@In_2_O_3_ PHTs	23.2	370	5000	7/5	This work

## Data Availability

All data are included in the paper.
